# Environmental Candida auris and the Global Warming Emergence Hypothesis

**DOI:** 10.1128/mBio.00360-21

**Published:** 2021-03-16

**Authors:** Arturo Casadevall, Dimitrios P. Kontoyiannis, Vincent Robert

**Affiliations:** a Department of Molecular Microbiology and Immunology, Johns Hopkins Bloomberg School of Public Health, Baltimore, Maryland, USA; b Division of Internal Medicine, The University of Texas MD Anderson Cancer Center, Houston, Texas, USA; c Westerdijk Fungal Biodiversity Institute, Utrecht, Netherlands

**Keywords:** *Candida*, global warming, virulence

## Abstract

Global warming was proposed to be a contributing cause for the nearly simultaneous emergence of different clades of Candida auris as a nosocomial pathogen in different continents. The global warming emergence hypothesis posits that C. auris existed in the environment prior to its clinical recognition and became pathogenic for humans because of thermal adaptation in response to climate change. The isolation of C. auris from two sites in the remote Andaman Islands establishes it as an environmental organism, a necessary condition for the hypothesis. The observation that one environmental isolate grew slower at mammalian temperatures than clinical strains is consistent with the notion that their ancestor recently adapted to higher temperatures. The knowledge that C. auris can be recovered from the environment should prompt additional searches to define its ecological niches, and the analysis of future environmental isolates will provide evidence for validating or refuting the global warming emergence hypothesis.

## Abstract (precis)

Global warming was proposed to be a contributing cause for the nearly simultaneous emergence of different clades of Candida auris as a nosocomial pathogen in different continents. The global warming emergence hypothesis posits that C. auris existed in the environment prior to its clinical recognition and became pathogenic for humans because of thermal adaptation in response to climate change. The isolation of C. auris from two sites in the remote Andaman Islands establishes it as an environmental organism, a necessary condition for the hypothesis.

## COMMENTARY

In their recent article in *mBio*, Arora et al. (1) describe the first environmental isolations of Candida auris from a sandy beach and a tidal swamp in the Andaman Islands. This landmark discovery is crucial for understanding the epidemiology, ecology, and emergence of C. auris as a human pathogen.

C. auris burst into the medical consciousness in 2011 to 2012 when it was simultaneously observed to cause serious disease in patients in Asia, Africa, and South America with clinical strains belonging to phylogenetically distant clades (2–4). Adding to the problem, C. auris was disconcertingly resistant to major antifungal drugs (4). This history poses several vexing questions that have been the subject of several recent articles (2, 4, 5). Where did C. auris come from? How does an organism emerge to become a human pathogen independently in geographically separated sites? What is driving the emergence of virulence in C. auris?

The isolation of C. auris from two environmental sites in the Andaman Islands establishes that this fungus can have an environmental niche and suggests an original environmental source for clinical isolates. Survival in the wild is important because unlike mammalian hosts, that environment is a place where microorganisms experience large temperature fluctuations, extremes of humidity, and predation by other microbes such as amoeba, which can select for traits that contribute to virulence (6). In fact, C. auris was recently reported to be preyed upon by two free-living amoeba and to proliferate when exposed to protozoal supernatants, which led the authors to suggest natural habitation in aquatic reservoirs (7). Hence, the capacity to survive in the environment implies hardiness to biotic predators and physical stresses such as insolation, temperature variation, and changes in humidity, each of which is likely to be present in both the rocky beach and tidal salt marsh sites from which C. auris was isolated in the study by Arora et al. (1).

The isolates from the beach resembled clinical isolates and may have reached that site from human sources through one discrete introduction, which is supported by their clonal relatedness. It is conceivable that they could have been carried by a person or animal to that site after acquisition from a contaminated health care site. However, the two isolates from salt marsh are particularly interesting because that site is remote from human activity, and they are different from each other. Most interesting is isolate VPCI/E/AN/176/20, which is significantly different from clinical isolates in being more susceptible to antifungal agents and growing slowly at 37 and 42°C. These differences suggest that VPCI/E/AN/176/20 is wilder, and perhaps closer to wild C. auris strains that inhabit environmental sites without passage through human hosts or facilities.

Over a decade ago, some of us proposed that the relative paucity of mycotic diseases for mammals reflected the rarity of fungal species that could survive and replicate at mammalian temperatures (8). That observation combined with a high likelihood that some fungal species could adapt to global warming by increasing their thermal tolerance led to the concern that global warming would bring new fungal diseases as adaptation to warmer temperatures defeated the protection provided by endothermy (9). For C. auris, its simultaneous appearance in diverse regions of the globe led to the proposal that this was the first example of a fungal species with pathogenic potential breaking through the mammalian endothermy barrier as a consequence of adaptation to climate warming (10). This idea is referred to as the global warming emergence hypothesis.

Although validating the global warming emergence hypothesis for C. auris will require considerable research, invalidating it is easier. For example, if C. auris had been found to be exclusively associated only with an endothermic animal such as an avian or mammalian species without an environmental reservoir, such that it was already thermally tolerant before its clinical appearance, that would obviate the need to invoke global warming as the cause for acquiring the capacity to grow at human temperatures. Hence, a necessary, although not a sufficient, requirement for the validity of this hypothesis is that C. auris has an environmental niche, and its discovery in the Andaman Islands provides a critical link.

Another finding that would support the global warming emergence hypothesis for C. auris would be the finding of closely related strains in the environment that have not fully adapted (at least for now) to grow at mammalian temperatures. Here the findings of Arora et al. (1) also provide supportive evidence in the form of recovering isolate VPCI/E/AN/176/20, which has reduced thermal tolerance relative to clinical C. auris strains. This isolate also has less intrinsic antifungal resistance, which could lend credence to the hypothesis that drug resistance in clinical isolates isolated in other parts of the world emerged from selection by environmental use of such drugs in agriculture (11). If VPCI/E/AN/176/20 is indeed closer to the wild ancestor of clinical strains, it suggests that wild C. auris has considerable intrinsic tolerance to higher temperatures such that adaptation to growth at mammalian temperatures could have required a relatively small jump in heat tolerance. Finding C. auris in a hot tropical climate fits with the notion that human fungal pathogens are more prevalent in equatorial regions where constant warmth has already led to thermal adaptation such that its maximum temperature tolerance is closer to mammalian temperatures (9).

The discovery of C. auris in the environment will encourage others to look for additional ecological sites harboring this organism. Of particular interest is to identify many more wild strains and characterize them for thermal tolerance and antifungal drug susceptibility. Clinical strains are primarily recovered from cases of nosocomial invasive infection where patients are infected with health care setting-adapted strains that may be quite different from their free-living environmental ancestors. Validating or refuting the global warming emergence hypothesis for C. auris will require a deeper exploration of the environmental isolates and closely related species. Support for the hypothesis would consist of finding more C. auris isolates with borderline mammalian thermotolerance and perhaps that these isolates can be adapted to grow at higher temperatures through laboratory selection for growth at higher temperatures.
